# Electroconductive Nanofibrous Scaffolds Enable Neuronal Differentiation in Response to Electrical Stimulation without Exogenous Inducing Factors

**DOI:** 10.3390/bioengineering10121438

**Published:** 2023-12-18

**Authors:** Nika Ranjbar, Behnaz Bakhshandeh, Cristian Pablo Pennisi

**Affiliations:** 1 Department of Biotechnology, College of Science, University of Tehran, Tehran 14155-6455, Iran; 2 Regenerative Medicine Group, Department of Health Science and Technology, Aalborg University, DK-9260 Gistrup, Denmark

**Keywords:** electrical induction, nanofibrous scaffold, neural differentiation, neural tissue engineering, mesenchymal stem cells

## Abstract

Among the various biochemical and biophysical inducers for neural regeneration, electrical stimulation (ES) has recently attracted considerable attention as an efficient means to induce neuronal differentiation in tissue engineering approaches. The aim of this in vitro study was to develop a nanofibrous scaffold that enables ES-mediated neuronal differentiation in the absence of exogenous soluble inducers. A nanofibrous scaffold composed of polycaprolactone (PCL), poly-L-lactic acid (PLLA), and single-walled nanotubes (SWNTs) was fabricated via electrospinning and its physicochemical properties were investigated. The cytocompatibility of the electrospun composite with the PC12 cell line and bone marrow-derived mesenchymal stem cells (BMSCs) was investigated. The results showed that the PCL/PLLA/SWNT nanofibrous scaffold did not exhibit cytotoxicity and supported cell attachment, spreading, and proliferation. ES was applied to cells cultured on the nanofibrous scaffolds at different intensities and the expression of the three neural markers (Nestin, Microtubule-associated protein 2, and β tubulin-3) was evaluated using RT-qPCR analysis. The results showed that the highest expression of neural markers could be achieved at an electric field intensity of 200 mV/cm, suggesting that the scaffold in combination with ES can be an efficient tool to accelerate neural differentiation in the absence of exogenous soluble inducers. This has important implications for the regeneration of nerve injuries and may provide insights for further investigations of the mechanisms underlying ES-mediated neuronal commitment.

## 1. Introduction

Currently, millions of people suffer from nerve injuries and neurodegenerative disorders, experiencing severe and unrelenting pain or the total loss of sensation in the body, while nerve regeneration remains challenging in the clinic [1]. Much effort has been devoted to developing innovative approaches to enhancing neural tissue engineering (NTE) [2,3], using a combination of cells, biomaterials, and biophysical/biochemical induction factors to replace impaired tissues [4,5].

Advances in stem cell technology combined with tissue engineering (TE) have opened new avenues for the regeneration of the nervous system [6,7]. It is known that different types of stem cells can contribute to the regeneration of neural tissue, including embryonic, adult, and induced pluripotent stem cells. Mesenchymal stem cells (MSCs), also referred to as mesenchymal stromal cells, are multipotent adult stem cells that have the capacity for self-renewal and multilineage differentiation, making them a great candidate for regenerative medicine [8,9]. Previous studies have reported the beneficial effects of MSC-based therapies in the treatment of various diseases such as neurological disorders, cardiac ischemia, diabetes, and musculoskeletal diseases [10]. While stem cell technology offers promising opportunities, it also presents challenges such as low survival rates, uncontrolled differentiation, and potential tumorigenicity [11]. Therefore, NTE currently focuses on the efficient and precise control of neuronal differentiation, including the incorporation of biomaterial scaffolds and the use of biochemical or physical triggers [12,13,14].

In the field of biomaterials, various natural and synthetic polymers have been developed for use as TE scaffolds [15]. Electrospinning is one of the most commonly used methods for nanofibrous scaffold fabrication using natural or synthetic materials [16]. Electrospun nanofibers have gained substantial attention because they mimic the architecture of natural fibrils in the extracellular matrix (ECM) providing a supportive environment for the growth, proliferation, and differentiation of stem cells [16,17].

Owing to the rapid degradation of natural materials in vivo and their relatively low mechanical strength, FDA-approved synthetic materials, including polycaprolactone (PCL) and poly L-lactic acid (PLLA), have been extensively studied in biomedical applications. Their properties, such as their biocompatibility, biodegradability, mechanical properties, porosity, and rigidity, can be modified to mimic those of natural tissues [18,19,20]. PCL- and PLLA-based electrospun nanofibers can also enhance neuronal proliferation and stimulate neurite outgrowth, making them suitable for applications in regenerative medicine [21,22]. However, synthetic materials may require surface functionalization because of the lack of integrin-binding molecules [21].

Since the neurons of the nervous system are naturally capable of transmitting electrical signals and are very sensitive to electrical stimuli, the development of electrically conductive biomaterials has attracted attention. These scaffolds serve as a conduit through which electrical stimulation (ES) can be effectively administered [23]. Thus, increasing scaffold conductivity through the incorporation of electroconductive carbon-based materials such as CNTs, graphite, and graphene into the scaffold can positively promote neural commitment [24]. Carbon nanotubes (CNTs) exhibit exceptional properties, including a large surface area, low electrical resistance, high thermal conductivity, mechanical strength, and excellent charge injection, making them promising candidates for various biomedical applications such as drug delivery, biosensing, and TE. In addition, CNTs have been broadly investigated as interesting materials for NTE, because they support neuronal adhesion and growth [25,26]. Single-walled nanotubes (SWNTs), as a single sheet of graphite, show low electrical resistance (resistivity = 1 μΩ·cm) and mechanical strength (Young’s modulus = 0.6–1.25 TPa) [27]. They are fundamentally pure carbon polymers, with each carbon in the lattice covalently bound to only three neighboring carbon atoms. The highly symmetric beam and truss structures formed by covalently bound carbon atoms make them exceptional nanomaterials for scaffold synthesis with high stability, strength, and flexibility [28].

Among the different biochemical and biophysical inducers that enhance stem cell-based NTE, ES has proven to be a promising tool both in vitro and in vivo [29,30]. In nervous tissue, endogenously generated electric fields (EFs) play pivotal roles in various biological phenomena, including reducing pain, nerve development, and regeneration through the transmission of electrical impulses [31,32,33]. ES can induce favorable biochemical and physiological responses, such as cell membrane depolarization and the alteration of membrane potential, and influence membrane protein roles, such as membrane receptors and ion transport channels. Likewise, ES affects several intracellular events, including the increased secretion of growth factors, triggering proliferation signaling pathways, increasing intracellular calcium (Ca^2+^), and interfering with the cell cycle and p53 levels [30,34]. Thus, determining whether electrical signals alone are capable of inducing the neural phenotype in the absence of exogenous inducing factors would be of great help for further investigation. 

Different types of exogenous EFs, including direct current (DC), alternating current (AC), and pulsed electric fields (PEFs), have been applied to cells or tissues in vitro. In addition, there are other non-invasive ways of using EFs on cells, such as capacitive and inductive coupling. Among the different methods, DC-EFs are particularly interesting because of their proper influence on the morphology, migration, proliferation, and differentiation of various cell types [25,35]. Typically, experimental DC-EF setups are made of Petri dishes or cell plates with electroconductive electrodes placed directly in the culture media. The intensity, frequency, voltage, duration, electrical current pattern, and electroconductive material used vary depending on the cell type and experimental setup, which contributes to slight variations in the effects of ES on neurogenesis across different studies. Therefore, determining the optimal setting for applying ES to different cells in NTE is challenging. Recent studies have advocated combinatorial strategies using conductive substrates and ES to accelerate functional nerve regeneration [18,33]. Although soluble biochemical inducers, including growth factors, are widely used to regulate stem cell fate, some drawbacks, such as high cost, off-target activities, fast degradation, and enzymatic inactivation under physiological conditions, possible toxicity, and difficulty in determining optimal release kinetics, raise serious concerns for clinical applications [36,37]. Thus, we focused on the potential effects of biophysical induction factors, such as ES, without using any chemical factors on lineage commitment.

Based on these considerations, in this study, a PCL/PLLA/SWNT scaffold was fabricated as a potential platform for neural differentiation and characterized using different methods. The cell adhesion, proliferation, and possible cytotoxic effects of the scaffold on the PC12 cell line and rat bone marrow mesenchymal stem cells (BMSCs) were investigated. The PC12 cells were selected as a model cell line because they are commonly used for NTE research, and there is abundant literature comparing the results of electrical induction [18,38]. Then, the effect of two electrical induction models (100 mV/cm for 10 min/day and 200 mV/cm for 20 min/day) to promote neural differentiation in seeded PC12 cells and BMSCs on PCL/PLLA/SWNT scaffolds was investigated using qPCR analyses of specific neural markers [39,40,41].

## 2. Materials and Methods

Poly-ε-caprolactone (PCL, CAS number 24980-41-4, average Mn 80,000) was obtained from Sigma Aldrich (Darmstadt, Germany), and single wall carbon nanotube (SWNT) N 70% (TGA) (7,6) chirality, ≥90% carbon basis (≥77% as carbon nanotubes), 0.83 nm average diameter/CAS number 308068-56-6) were purchased from Sigma Aldrich. Poly L-lactic acid (PLLA, CAS number 764698, average Mn 20,000, PDI ≤ 1.1) (grade: 2500 HP) was supplied by Natureworks LLC (Plymouth, MN, USA).

Dulbecco’s Modified Eagle’s Medium (DMEM) high glucose, Trypsin/EDTA 0.25%, penicillin/streptomycin, fetal bovine serum (FBS), and phosphate-buffered saline (PBS) were purchased from Gibco (Dreieich, Germany). Trypan blue was purchased from Sigma-Aldrich (St. Louis, MO, USA). 3-(4,5-dimethylthiazol-2-yl)-2,5-diphenyltetrazolium bromide (MTT) powder, dimethyl sulfoxide (DMSO), ethanol, chloroform, dimethyl formamide (DMF), and isopropanol were purchased from Merck (Darmstadt, Germany) and used without further purification.

### 2.1. Nanofibrous Scaffold Fabrication

In brief, 20% (*w*/*v*) PCL polymer solution was prepared by dissolving polymer granules in a 9:1 ratio of chloroform and DMF solvents and stirred for about 2 h on a magnet stirrer [42]. SWNT powder was dispersed in the same solvents in an ultrasound sonicator for 1 h to make a black and concentrated solution. Then mono-dispersed SWNT solution was added to the PCL solution and mixed well by stirring for more than 2 h. Simultaneously, PLLA was mixed with a 6:1 ratio of chloroform and DMF solvents to yield a total polymer weight of 6% (*w*/*v*) [43,44]. After PCL/SWNT and PLLA solutions were stirred sufficiently to obtain homogeneous solutions, 5 mL of prepared solutions were separately placed in plastic syringes with 22-gauge needles connected to a high-voltage power supply. The collector was covered with aluminum foil for easy removal of the scaffolds. 

The nanofibers were produced under the following conditions by the two-nozzle electrospinning method. All scaffolds were fabricated using a 20 KV voltage at the nozzle tip, 0.5 mL/h feeding rate with 12 cm needle-collector distance on the rotating drum (300 rpm) with 8 cm scan rate. Due to the hydrophobic nature of PCL and PLLA polymers, the surface of the nanofibrous PCL/PLLA/SWNT scaffold was modified using plasma treatment with a low-frequency plasma generator for 30 s with a cylindrical quartz reactor (Diener Electronics, Ebhausen, Germany) to facilitate cell attachment and adhesion (0.5 mbar pressure). All experiments were performed using plasma-treated PCL/PLLA/SWCNT nanofibrous composites. 

For biological investigations, scaffolds were sterilized under 1 h of UV radiation and immersed three times in a 70% aqueous ethanol solution for 20–30 min. After the ethanol solution evaporated in the air, the samples were rinsed with sterile PBS buffer three times to remove the residual ethanol. 

The structural morphology of electrospun nanofibers was evaluated using SEM (Seron Technologies, model AIS 2100, Gyeonggi-do, Republic of Korea). The fiber diameters of the electrospun nanofibers were also measured using SEM micrographs and image analysis software Image J version 1.53k (National Institutes of Health, Bethesda, MD, USA).

### 2.2. Nanofibrous Scaffolds Characterization

#### 2.2.1. Fourier-Transform Infrared Spectroscopy (FTIR)

In order to confirm the expected bonds and functional groups, FTIR Spectroscopy (Perkin Elmer, Frontier, Waltham, MA, USA) was performed over a range of 400–4000 cm^−1^.

#### 2.2.2. Degradation Behavior

The degradation behavior of the scaffolds was studied by measuring the percentages of swelling rate and weight loss during 40 days of incubation [45]. The swelling behavior of the scaffolds was assessed in PBS buffer using a gravimetric method. In brief, the scaffolds were cut into 1 × 1 cm^2^ pieces, and then the pre-weighted dry samples (*Wd*) were dipped in the PBS buffer (pH = 7.4) at 37 °C. After 1, 10, 20, 30, and 40 days, the samples were taken out from the PBS and weighed again after wiping the excess buffer off the samples onto a filter paper (*Ww*). The ratio of swelling was calculated using the following Formula (1):

(1) $$swelling\;ratio\;(\%)=\frac{Ww-Wd}{Wd}\times 100$$

where *Ww* is the wet weight, and *Wd* is the dry weight. 

The degradation of all scaffold types was evaluated by measuring the weight loss of scaffold specimens (1 × 1 cm^2^) during 40 days of incubation in PBS buffer (pH = 7.4) at 37 °C. The initial weight of samples (*Wi*) was recorded, and the PBS solution was discarded after its pH was recorded at designated time points (1, 10, 20, 30, and 40). After that, the specimens were washed three times with double deionized water, placed in an oven at 37 °C for about 4 h, and finally reweighed (*Wd*). The degradation rate of the scaffolds was determined using the following Equation (2):

(2) $$Degradation\;rate\;(\%)=\frac{Wi-Wd}{Wi}\times 100$$

where *Wi* is the initial weight, and *Wd* is the dry weight. All measurements were performed in triplicate [46,47].

#### 2.2.3. Water Contact Angle (WCA) Assay 

To assess the hydrophilicity of the mats, WCA at the surface of scaffolds (PCL, PCL/PLLA, PCL/PLLA/SWNT, and plasma-treated PCL/PLLA/SWNT) were investigated using a Data Physics Contact Angle Goniometer (OCA-200, DataPhysics Instruments GmbH, Filderstadt, Germany). Briefly, each sample was placed on the measuring stage, then a drop of 3 μL of deionized water was placed onto different locations of samples at 25 °C. Measurements of WCA were carried out using Image J software, version 1.53k. The reported WCA values were the average of at least three independent measurements.

#### 2.2.4. Mechanical Properties

The mechanical behavior of fabricated scaffolds was studied using Instron 3367 tensile testing device (STM20, SANTAM, Tehran, Iran). Samples with dimensions of 5 × 30 mm^2^ were punched from a single electrospun sheet. Samples were tested at 25 °C with 10 mm/min loading velocity (3 replicates).

#### 2.2.5. Conductivity Measurement

The resistance of polymer films was evaluated using the 4-point probe method [48]. The scaffold resistance was calculated using following equation:

(3) $$R=\rho \frac{L}{A\;}$$

where *R* is sheet resistivity (Ω), ρ is specific resistivity (Ω cm), *L* is fiber mat length (cm), and *A* is the cross-sectional area (cm^2^).

Also, according to Equation (4), the conductivity was calculated.

(4) σ=L/RA

where *σ* is specific conductivity (S/cm), *R* is sheet resistivity (Ω), *L* is fiber mat length (cm), and *A* is the cross-sectional area (cm^2^).

### 2.3. Cell Culture and Visualization

Both PC12 cells and BMSCs (derived from *Rattus norvegicus*) were purchased from Bonyakhteh Company, Tehran, Iran, and were routinely cultured in high glucose DMEM medium with 10% FBS serum, supplemented with 1% penicillin/streptomycin antibiotic. All cells were maintained in a humidified atmosphere containing 5% CO_2_ at 37 °C. The culture media were changed every second day. 

To monitor cell adhesion, after the 3 and 5 days of culture, cells were washed with PBS to remove dead cells, fixed with 2.5% aqueous glutaraldehyde solution for 3 h, and washed, respectively, with aqueous ethanol solution (50–100% *v*/*v*). For visualization of cell nuclei, the cells were incubated with 4′,6-diamidino-2-phenylindole (DAPI) stain for 10 min at room temperature. Image acquisition was performed using a wide-field fluorescence microscope (Olympus, Tokyo, Japan) using an excitation wavelength of 450 nm. For SEM analysis, after air drying, the specimens were mounted onto a stub and coated with gold. After 15 min waiting to remove residual water, samples were observed under SEM (Seron Technologies).

### 2.4. Cell Proliferation Assay

The proliferation of PC12 cells and BMSCs seeded on electrospun mats was determined using colorimetric MTT assay. Sterilized electrospun PCL/PLLA/SWNT scaffolds were placed at the bottom of the wells of 48-well tissue culture plates. The cells were harvested at 85–90% confluency using 0.25% trypsin-EDTA. After enzymatic digestion, cells were cultured at a density of 3000 cells/well density on the scaffolds. Similar numbers of cells were cultured on 2D tissue culture as control. The plate was incubated at 37 °C in the 5% CO_2_ incubator, and the cell media were changed every two days. After 1, 3, and 7 days of culture, 50 μL MTT of solution (5 mg/mL) was added to each well and incubated at 37 °C for 4 h. Subsequently, the medium was gently removed, 150 μL of DMSO solution was added to each well, and the plate was shaken for 30 min at room temperature to solubilize formazan crystals. The aliquots were then pipetted into the wells of a 96-well plate and the optical density was determined using a spectrophotometric microplate reader (Biotek, Winooski, VT, USA) at 570 nm.

### 2.5. Electrical Induction

Circle-shaped sections of scaffolds (A ≈ 3.5 cm^2^) were fixed to the bottom of the custom-built 12-well cell culture plate and incubated for 2–3 h with culture media. When the cells reached 90% confluency, they were digested with 0.25% trypsin-EDTA solution, washed, and resuspended in DMEM high glucose with 10% FBS. Next, 100,000 cells were cultured on the PCL/PLLA/SWNT scaffolds in each well and incubated under EF-free conditions for 24 h to allow attachment. Experiments were performed based on previous studies by selecting an optimum field strength of 100 mV/cm in a stimulation cycle of 10 min/day [39]. We also investigated the impact of increasing the field strength and induction duration by applying 200 mV/cm in a stimulation cycle of 20 min/day for 7 days [40,41]. A picture of the actual setup for delivering electrical stimulation is shown in the Supplementary Figure S1 (Supplementary Materials). Two 316 stainless-steel electrodes were placed at opposite ends of the nanofibrous membranes in a custom-built electrical tissue culture plate. The electrodes were connected to a function generator capable of supplying direct current (DC) (Function/Arbitrary Waveform Generator, GPS Lt, London, UK). During induction time, the tissue culture plates were placed in a 5% CO_2_ incubator at 37 °C. After applying EF, the culture medium was changed with fresh media.

### 2.6. Transcriptional Analyses of Neural Marker Genes

Total RNA was extracted using guanidine/phenol reagent (RNX-Plus, SinaClon, Tehran, Iran, CAS number EX6101) and quantified by spectrophotometry. Then, cDNA was prepared from 2 μg of total RNA using the M-MLV Reverse transcriptase enzyme, random hexamer, and oligo dT primers (SinaClon) according to the manufacturer’s instructions. RT-qPCR was performed to evaluate the expression of three neuronal markers, Nestin (*NES*), β Tubulin-3 (*TUBB*), and Microtubule-associated protein 2 (*MAP-2*) in rat-PC12 cells and BMSCs. RT-qPCR was performed using Rotor-Gene Q, QIAGEN (Hilden, Germany) with Real Q Plus 2x SYBR Green Master mix (without ROX, Ampliqon, Odense, Denmark) with the following qPCR program: an initial denaturing step of 15 min at 95 °C, followed by 45 cycles (95 °C for 15 s, 60 °C for 20 s, and 72 °C for 30 s), and a final extension step for 1 min at 72 °C. Relative gene expression levels were determined using the ΔΔCt method, whereby the target genes were normalized to the hypoxanthine phosphoribosyl transferase 1 (*HPRT1*) housekeeping gene as an endogenous reference and then compared with the control. Normalized fold differences in the expression of neural marker genes for cells seeded on PCL/PLLA/SWNT with or without electrical stimulation were compared with those cultured on TCP. The data for the different time points (days 3 and 7) were normalized to the corresponding day 0 values, except for *TUBB* and *MAP-2* expression in BMSCs, where the data were normalized to the day 7 values on TCP. The primers used in this study are listed in Table 1.

### 2.7. Statistics

Statistical analysis was performed using the Prism statistical package GraphPad Software, version 8 (San Diego, CA, USA) and data were presented as the means ± SD (standard deviation). To ascertain significance, a one-way ANOVA with Tukey’s Post Hoc correction test was used. Statistical significance was determined at a limit of *p* < 0.05 from three independent experiments conducted at least in duplicates.

## 3. Results and Discussion 

### 3.1. Nanofibrous Scaffolds Characterization

PCL and PLLA are synthetic polymers with appropriate biocompatibility and biodegradability that are extensively used in TE applications [49] and were chosen in the present study owing to their favorable characteristics. The physicochemical characteristics of the nanofibrous electrospun scaffolds were evaluated using FTIR spectroscopy, scanning electron microscopy, WCA, biodegradation behavior, conductivity, and mechanical measurements.

#### 3.1.1. Electrospun Nanofibers Morphology

**Figure 1:**
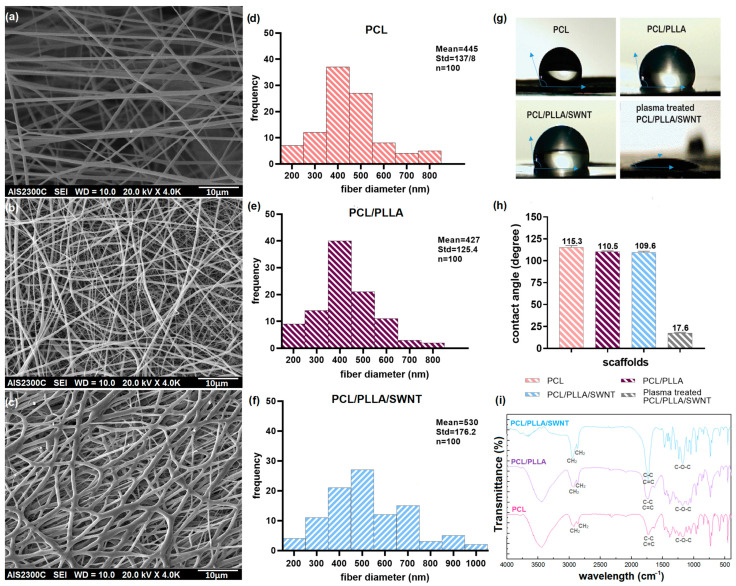
(**a**–**c**) SEM micrographs of PCL, PCL/PLLA, and PCL/PLLA/SWNT nanofibrous composites. Scale bars = 10 µm. (**d**–**f**) The fiber diameter distribution graphs of nanofibrous composites. (**g**,**h**) WCA measurement of electrospun PCL, PCL/PLLA, and PCL/PLLA/SWNT and plasma-treated PCL/PLLA/SWNT nanofibers. After a few seconds, the droplet on plasma-treated scaffolds was absorbed and WCA quickly reduced to zero. (**i**) FTIR spectra of nanofibrous composites.

SEM micrographs of PCL, PCL/PLLA, and PCL/PLLA/SWNT nanofiber membranes are shown in Figure 1, which show uniform and smooth nanofibers without bead defects. Furthermore, the investigated scaffolds had fibrous and porous structures with interconnected pores, providing an appropriate platform for essential nutrients and oxygen diffusion [50,51]. The electrospun neat PCL nanofibers had an average diameter of 445 ± 138 nm. The average diameter of the PCL/PLLA and PCL/PLLA/SWNT nanofibers were 427 ± 125.4 nm and 530 ± 176.2 nm, respectively. Previous studies have shown that nanoscale fibers increase the surface area for better cell adhesion, growth, and proliferation [20,52]. It was demonstrated that adding CNTs led to an increase in the diameter of the nanofibers, probably because of the high viscosity of the nanofibers after using CNTs [53]. Solution viscosity is one of the most important and influential factors affecting the morphology and diameter of the nanofibers. We did not exceed the CNTs concentration to minimize cytotoxic effects and avoid the potential for CNTs agglomeration within the nanofiber. Thus, minimal CNTs agglomeration was observed within the fiber constructs (Figure 1c). This result indicates the adequate dispersion of CNTs within the polymer mixture [54,55].

#### 3.1.2. WCA of Electrospun PCL/PLLA/SWNTs Nanofibers

The hydrophobic or hydrophilic characteristics of the nanofibrous scaffolds were investigated using a WCA measurement. Then, the measured angles were determined using Image J software and reported as a mean and a standard deviation. The measured contact angle of all three scaffolds, PCL, PCL/PLLA, and PLC/PLLA/SWNT, was above 100 degrees, indicating the hydrophobicity of their surfaces [56]. Due to the importance of the hydrophilicity of the surface for cell adhesion and attachment, the PCL/PLLA/SWNT scaffolds were modified via plasma surface modification. Plasma treatment is a well-known post-processing method that increases hydrophilicity and cell adhesion. The WCA was recorded at around 18 degrees after a few seconds and quickly reduced to zero (Figure 1g,h). Therefore, surface modification with plasma renders the scaffold completely hydrophilic, improving cell adhesion [57,58]. Consequently, all experiments were performed using plasma-treated PCL/PLLA/SWCNT nanofibrous composites.

#### 3.1.3. FTIR of Electrospun PCL/PLLA/SWNTs Nanofibers 

The FTIR spectra of the PCL, PCL/PLLA, and PCL/PLLA/SWNT nanofibrous composites represent the chemical structures of the designed scaffolds in 400–4000 cm^−1^ (Figure 1i). The figure shows absorption peaks at 2924, 2860, 1728, 1294, 1240, and 1175 cm^−1^ in PCL spectra. The peaks at 2924, 2860, and 1728 cm^−1^ correspond to asymmetric and symmetric stretching vibrations of the C–H bond in the methylene (CH_2_) group and carbonyl C=O stretching vibrations, respectively. The peaks at 1294 and 1240 cm^−1^ represent the C–O and C–C stretching in the crystalline phase of PCL, and the peak at 1176 cm^−1^ represents the asymmetric and symmetric C–O–C stretching [59]. 

Due to the structural uniformity of the two polymers, aliphatic polyesters, there was no change in the spectra of the blend polymers. In the spectrum of PCL/PLLA, the peaks at 2931 and 2865 cm^−1^ correspond to the C–H vibrations present in the methylene and methyl groups, and 1742 cm^−1^ represents the characteristic carbonyl (C=O) peaks of PCL and PLLA. The absorption at 1294 and 1239 cm^−1^ represents the C–O and C–C stretching of PCL, respectively. The peak at 1182 cm^−1^ represents the C–O–C linkage present in PCL and PLLA [60]. These observations clearly confirmed the presence of PCL and PLLA in PCL/PLLA composite nanofibers. The PCL/PLLA/SWNT FTIR spectrum has absorption peaks of 2949, 2867, 1733, 1294, 1240, and 1170 cm^−1^, similar to the spectra of the two polymers, PCL and PCL/PLLA. Because of the small concentration of CNTs in the polymer nanofiber (1% wt.), no changes in the position of the peaks were observed. A comparison between spectra of neat PCL and PCL/PLLA indicated that the bands assigned to SWNT did not shift their wavenumbers in scaffolds. However, the peak intensity at 1733 cm^−1^, which is related to carbonyl C=O stretching, significantly increased [61,62]. All these observations indicate the presence of PCL, PLLA, and SWNTs in PCL/PLLA/SWNT nanofibrous scaffolds.

#### 3.1.4. Degradation Behavior of Electrospun PCL/PLLA/SWNTs Nanofibers

**Figure 2:**
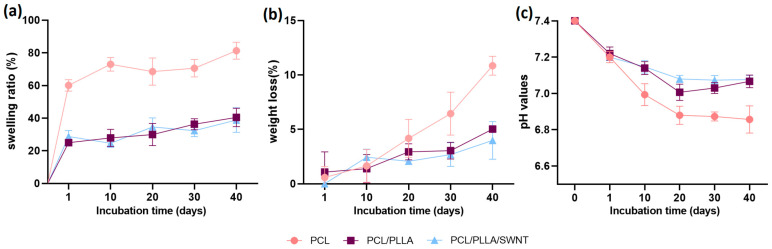
In vitro biodegradation behavior (**a**) Swelling ratio, (**b**) Weight loss, (**c**) Change in pH and of PCL, PCL/PLLA, and PCL/PLLA/SWNT nanofibrous scaffolds (Error bars show SD. Sample size n = 3).

The degradation behavior of the scaffolds was assessed by measuring the weight loss and swelling rate during 40 days of incubation in PBS solution (pH = 7.4) for different periods (1, 10, 20, 30, and 40 days) at 37 °C (Figure 2). Due to the importance of nutrients and signal molecule diffusion in cell growth and proliferation, the appropriate swelling rate of the scaffold is an essential property of TE. However, an increase in water uptake might negatively affect the mechanical properties of the scaffold. Scaffold swelling depends on various physical factors such as electrospinning solution concentration, pH, surface area, and porosity [63,64]. The results showed that the highest swelling percentage was related to PCL nanofibers, and PCL/PLLA and PCL/PLLA/SWNT nanofibers showed relatively similar swelling ratios. Thus, 1% SWNTs did not significantly affect the water absorption rate (Figure 2a). The highest increase in absorption appeared at the beginning of the degradation period. PCL nanofibers showed swelling behavior up to 60% after being placed in PBS buffer solution at 37 °C for 1 day, and then the swelling rate gradually increased to 81% at the end of the period. The PCL/PLLA and PLC/PLLA/SWNT fibers showed about 40% water uptake [49,65,66]. The addition of PLLA to the scaffold considerably reduced the degree of swelling, probably due to the higher number of crystalline regions. Although the hydrophobic properties of PCL limit water diffusion due to the presence of long hydrocarbon chains, the amorphous nature of PCL makes water penetration relatively easier than that of PLLA nanofibers in the crystalline state. 

Biodegradable scaffolds have attracted more attention for TE applications because polymer chains with high molecular weights are hydrolyzed into non-toxic oligomers, providing the basis for the replacement and regeneration of natural tissue. PCL and PLLA polymers are aliphatic esters with relatively low degradation rates and have been used to fabricate devices implanted in the body. The time required for the complete degradation and absorption of a polymer principally depends on the molar weight, crystallinity, morphology, implant size, and chain type [66,67]. PCL degradation occurs during hydroxylation and the fragmentation of high molecular weight chains with or without enzymatic digestion in an aqueous environment or body fluids, and finally decomposes into water and CO_2_. All the nanofiber scaffolds showed a prolonged degradation rate, and the maximum weight loss was approximately 11% for PCL (Figure 2b). By adding PLLA, the scaffold degradation rate decreased to 6.5% compared with pure PCL, probably because of the presence of more crystalline regions in the PLLA structure than in PCL, which reduced the penetration of the buffer and medium into the scaffold pores. The in vitro degradation of PCL and PLLA nanofibers occurs in four steps: first, due to the penetration of PBS between the fibers, the scaffold swells, then the PCL and PLLA fibers are hydrolyzed, and their ester bonds are broken. Subsequently, the hydrolysis products are released into the environment, and eventually, the produced macromolecules disappear. This indicates that the degradation rate is specifically related to the exposure of ester bonds to water and depends on various factors. Owing to the inherent hydrophobic nature of PCL and PLLA, it is difficult for water molecules to penetrate polymer molecular chains during the early stages of degradation and perform the swelling stage [68]. Accordingly, the degradation rate was low, particularly during the first days of the experiment. 

Another explanation for the slow degradation rate, especially in the early timepoints, is the recrystallization of PLLA and the formation of spherulite structures [20,52,63]. In general, amorphous polymers degrade more easily than crystalline polymers do in aqueous environments. Weight loss in the first days of the experiment was slight and increased over time. Adding 1% SWNT to the scaffold did not significantly change the degradation process, but a negligible reduction in the degradation rate was observed. However, previous studies have shown that an increase in the carbon nanotubes content might lead to scaffold agglomeration and a decrease in the substrate integrity, which reduces the penetration of water and culture medium through the scaffold pores. Changes in the pH of PBS during degradation were also investigated (Figure 2c). Similar trends were observed in pH changes for all three scaffolds. The degradation of neat PCL nanofibers showed the greatest change in pH, decreasing to approximately 6.9. Since the degradation of PLLA and PCL release acidic species upon hydrolysis, the pH changes during the degradation process of PCL/PLLA and PCL/PLLA/SWNT nanofibers were almost the same and reduced from 7.4 initial value to around 7. The process of changes in the pH of the environment was consistent with the degradation process. 

#### 3.1.5. Mechanical Properties of Electrospun PCL/PLLA/SWNTs Nanofibers

**Figure 3:**
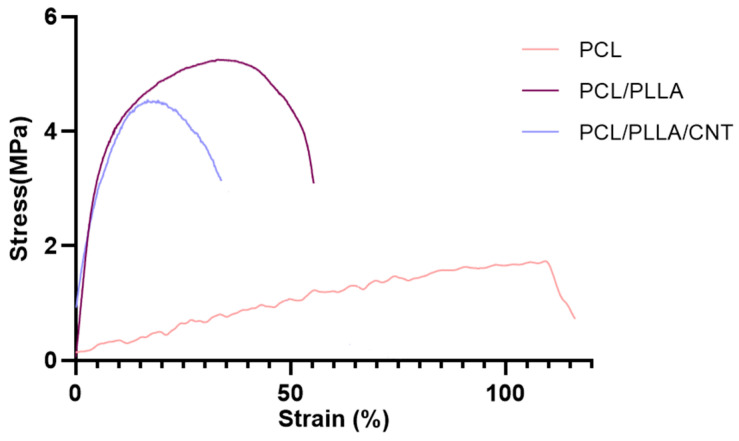
Representative tensile stress–strain curves of electrospun PCL, PCL/PLLA, and PCL/PLLA/SWNT nanofibrous scaffolds. (Sample size n = 3).

The mechanical behavior of the nanofibrous scaffolds was evaluated using tensile tests. The typical stress–strain curves are shown in Figure 3 and the mechanical parameters are listed in Table 2. Clearly, both the tensile strength and Young’s modulus of the PCL/PLLA and PCL/PLLA/SWNT scaffolds substantially increased compared with those of neat PCL [62,69]. PCL is a flexible polymer with a high level of toughness owing to its linear backbone structure without side groups or branch chains. After incorporating the PLLA nanofibers, the mechanical strength and Young’s modulus were considerably enhanced, whereas the elongation at break decreased adversely owing to the relatively high mechanical strength of PLLA [70,71]. 

The Young’s modulus of the scaffold in the presence of PLLA rose from 1.5 to 70.5 MPa compared with pure PCL, although it decreased to 39.5 MPa with the addition of CNTs, probably due to the possible agglomeration of the nanoparticles and the lack of monodispersity in the solution. The results showed the same trend for the change in tensile strength, which is in line with previous studies [72,73].

#### 3.1.6. Electrical Conductivity of Electrospun PCL/PLLA/SWNTs Nanofibers

Considering the nature of neural tissue and how it transmits signals, the electrical conductivity of substrates could improve nerve growth and thus increase nerve regeneration. The electrical conductivity of the nanofibers was tested using the 4-point probe method, and the results are listed in Table 1. No significant difference was observed between the conductivity of the PCL and PCL/PLLA scaffolds (both at approximately 0.03 μS/cm). The conductivity of the scaffolds was relatively weak, especially in a dry state. In this research, the conductivity of the scaffolds was evaluated in a dry state and after electrospinning. Studies have shown that conductivity measurement in the solution state before electrospinning leads to higher conductivity than those in the solid state. With the addition of CNTs, the electrical conductivity of the scaffolds approximately doubled (or the volume resistivity of the scaffold almost halved) indicating a good dispersion of CNTs [74,75]. According to the literature, different parameters affect the conductivity of CNT-containing nanofibers, such as the conductivity, size, and concentration of the nanoparticles and their dispersion in electrospinning solution. Although increasing the percentage of carbon nanotubes leads to a significant increase in conductivity, the use of CNT content must be limited due to cytotoxicity concerns; therefore, using controlled and limited amounts of these materials (less than 5%) has been suggested [76,77].

### 3.2. Biocompatibility of the Electrospun PCL/PLLA/SWNTs Nanofibers

**Figure 4:**
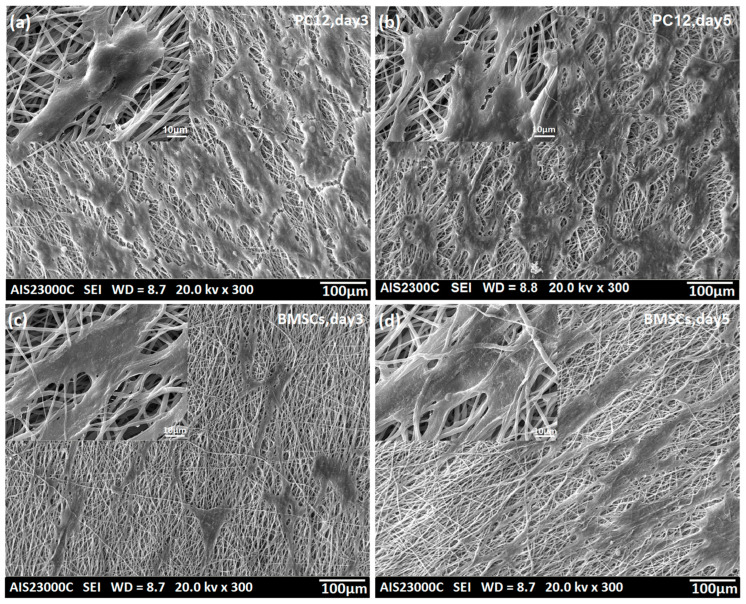
SEM images of the PC12 cells and BMSCs seeded on electrospun PCL/PLLA/SWNT nanofibrous scaffolds: (**a**,**b**) PC12 cells and (**c**,**d**) BMSCs on day 3 and 5 of culture. Scale bars for SEM micrographs = 100 and 10 µm.

**Figure 5:**
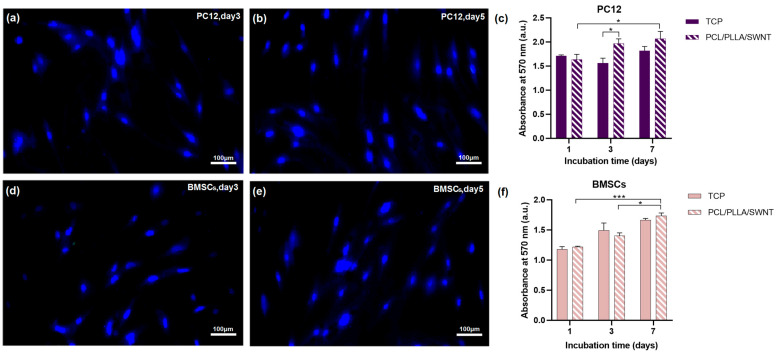
Fluorescence images (DAPI staining) and cell viability assay of PC12 cells and BMSCs grown on electrospun PCL/PLLA/SWNT nanofibers: (**a**,**b**) PC12 cells and (**d**,**e**) BMSCs on days 3 and 5 of culture. The nuclei were stained blue. Scale bars = 100 µm. (**c,f**) MTT assay graphs (error bars show SEM. Sample size n = 3. * Indicates *p* < 0.05, and *** *p* < 0.001).

The spreading and proliferation of PC12 cells and BMSCs on the surfaces of PCL/PLLA/SWNT nanofibrous scaffolds were investigated using SEM and DAPI staining images on days 3 and 5. Although PCL and PLLA have been successfully used in scaffold fabrication, cell adhesion to their surfaces is relatively weak due to the lack of functional groups. However, the figures revealed proper cell adhesion and the spreading of both cells on the plasma-modified scaffold, and the morphology of the cells changed on day 5, compared with day 3, from round or triangular to elongated and spindle-shaped (Figure 4 and Figure 5). 

The cell viability and potential cytotoxicity of the PCL/PLLA/SWNT scaffolds were investigated using a colorimetric MTT assay on days 1, 3, and 7. Previous studies revealed that both PCL and PLLA polymers possess exceptional biocompatibility and non-cytotoxicity [68,78]. The results indicated the scaffold had no toxic effect on the cells, and the proliferation of cells in both cell lines was enhanced. As shown in Figure 5, no statistical significance in cell viability was observed between cells cultured on the scaffold and those on TCP. Also, despite the potential toxicity and low degradability of CNTs, the low percentage of CNTs in the scaffold did not negatively impact cell viability and proliferation [79,80]. 

### 3.3. The Effect of Electrical Stimulation on Neural Differentiation 

**Figure 6:**
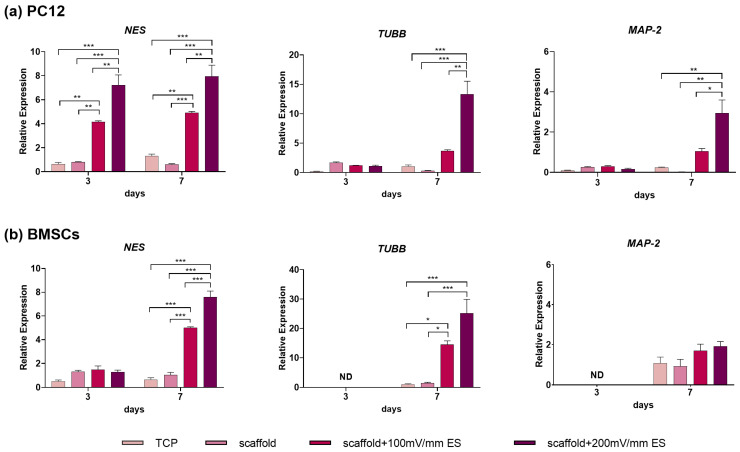
Relative expression of the neural progenitor marker *NES*, early neuronal marker *TUBB*, and neuron-specific protein marker *MAP-2* in (**a**) rat-PC12 cells and (**b**) BMSCs cultured on PCL/PLLA/SWNT scaffolds. The relative expression at days 3 and day 7 is shown for the four experimental conditions: TCP, scaffold, scaffold + 100 mV/cm ES, and scaffold + 200 mV/cm ES (Data represents means ± SEM of three independent experiments. ND: not detected. * Indicates *p* < 0.05, ** *p* < 0.01 and *** *p* < 0.001).

Having demonstrated the influence of the scaffold on the viability and proliferation of cells, we investigated the effect of ES on neural differentiation. We used Nestin (*NES*), a neural progenitor marker [81], β Tubulin-3 (*TUBB*), an early neuronal marker [82], and Microtubule-associated protein-2 (*MAP-2*), a late marker involved in neuronal differentiation [83]. The upregulation of these genes is typically associated with the induction of neural differentiation. Normalized fold differences in the expression of genes for cells seeded on scaffolds with (100 and 200 mV/cm) or without ES were compared with those cultured on TCPs. The results of RT-qPCR showed that the mRNA expression levels of the selected neural markers were upregulated in cells grown on the PCL/PLLA/SWNT composite scaffolds, especially after the application of electrical stimulation (Figure 6).

Various studies have employed diverse electrical stimulation protocols, including different field strengths, durations, and scaffold conductivities. Consequently, determining the optimal experimental conditions has become a matter of controversy. Ideally, an optimized external EF strength should induce changes in cell morphology and differentiation while minimizing adverse effects on cell survival [84]. Notably, physiologically relevant electric fields in natural tissues can reach strengths up to 2 V/cm [85]. Thrivikraman et al. identified 100 mV/cm during a 10 min stimulation cycle as the optimum EF for cells seeded on polyaniline (PANI) substrates [39]. These substrates transitioned from highly insulating to conducting due to doping with varying HCL concentrations (ranging from 10^−5^ to 1 M). It was emphasized that applying 500 mV/cm had detrimental effects on cell growth, especially on highly conductive substrates. However, field strengths lower than 100 mV/cm were too weak to induce significant changes in the hMSCs behavior In another study involving PC12 cells, EF from 0 to 100 mV/mm was applied for 2 h per day, indicating that applying 80 mV/mm for 2 h had no significant adverse effects on cell viability [18].

Furthermore, Grossemy et al. investigated the impact of DC ranging from 0.020 to 20 mV/mm for 2 h per day over 12 days on PC12 cells cultured on viscose-rayon scaffolds, both with and without gold components. The study revealed that there were no cytotoxic effects observed when up to 500 mV/mm was applied to nonconductive substrates or when up to 100 mV/mm was applied to scaffolds containing gold [86]. These studies collectively highlight the complexity of determining optimal ES parameters to promote desired cellular responses while avoiding detrimental effects on cell behavior and viability. Thus, we chose to apply 100 and 200 mV/cm for 10 and 20 min, respectively, considering that applying more intensity could be harmful to the cells.

The expression level of *NES* after 3 days in cells cultured on the scaffold under 100 and 200 mV/cm EF was around 4 and 7 times higher than its expression in cells without applying EF, respectively. While it remained relatively constant after 7 days, the expression of *MAP-2* and *TUBB* genes was considerably enhanced under ES, providing further evidence for the effective biophysical induction factor for promoting neural lineage commitment in the absence of exogenous growth factors. This is consistent with the previous literature whereby the exposure of PC12 cells to EFs improved neurite outgrowth [18,87,88]. In addition, the stronger EF (200 mV/cm) yields higher levels of all neural marker gene expression than 100 mV/cm EF.

For BMSCs, after 3 days of cell culture, all the samples showed no appreciable difference in *NES* expression. However, on day 7, in the cells in the “scaffold”, “scaffold + 100 mV/cm”, and “scaffold + 200 mV/cm” groups, the expression level increased by around 5.5, 5, and 8 times compared with those in TCP, which indicates a positive effect of the culture substrate and biophysical stimulation on the induction of the neural phenotype. On day 3, the transcriptional levels of *TUBB* and *MAP-2* were under detection limits in all the samples. Although the expression of *TUBB* on day 7 increased, especially under the 200 mV/cm condition (approximately 3.5-fold), *MAP-2* (as a late neural marker) expression did not significantly differ between the studied groups. Taken together, these results indicate that applying ES could substantially increase the neurogenic differentiation of BMSCs in vitro. 

The findings related to the gene expression analysis showed that the scaffold alone could not stimulate neural differentiation. However, in the presence of an external EF and by strengthening the EF intensity, the expression of neural markers considerably increased. Comparing the response to electrical induction in the two studied cell types (PC12 and BMSCs, which were isolated from *Rattus norvegicus*) indicated that, considering that the PC12 line is a neural progenitor, the expression of neural markers significantly enhanced after 7 days of treatment along with the intensity of the applied EF. Considering the upward trend of *NES* gene expression in BMSCs after 7 days of ES, it seems that better results might be achieved by examining the changes in gene expression over an extended period. 

Our results are consistent with those of previous reports indicating the effect of ES on neural lineage commitment. For instance, in one study, a biphasic square wave electrical induction on PC12 cells was performed to examine the influence of the conductive random and aligned PEO/PEDOT: PSS nanofiber mats. Their results showed an improvement in neurite outgrowth and the relative gene expressions of *NES*, *TUBB*, and *MAP-2* as well [89]. Applying 100 mV/cm for 10 min/day for 7 days to hMSCs seeded on highly conducting substrates revealed morphological changes and the increased expression of neural markers such as *NES* and *TUBB* [39]. In another study, they reported that the exposure of hMSCs to regular intermittent cycles of DC EF stimuli (100 mV/cm, 15 min/day for 7 days) with gold nanoparticles acquired morphological changes and higher neural-specific marker expression without using differential media [40]. 

Moreover, Matsumoto et al. found that electrically stimulated mouse-BMSCs (rectangular pulse 10 Hz, 100 mV, 2.0 ms, 30 min) have shown potential for neural differentiation. Their results demonstrated that, after ES, neurogenin2 gene expression increased through the β-catenin signaling pathway, which is involved in neural commitment and hinders astrocyte formation [90]. In addition, Pires et al. demonstrated that direct pulsed DCEF can successfully increase the neurite outgrowth and elongate neural stem cells, which were seeded on an electroconductive conjugated poly (3,4 ethylenedioxythiophene): polystyrene sulfonate (PEDOT:PSS) scaffold [91]. These reports show that the intensity of applying EF to cells could also increase neural differentiation. However, previous studies have indicated that increasing the intensity of the field above a certain threshold could inhibit cell growth and increase cell death [39,92]. The observed variations in sensitivity to ES between different cell types highlight the need to determine the optimal ES intensity and induction time to achieve the desired results before in vivo experiments. 

There are some inconsistencies between the various published studies, which is not unexpected given the different ES procedures. The current experimental in vitro paradigm for studying the effect of ES on cells involves the use of electrically conductive platforms in which an electronic current flows in close proximity to the cell–cell and cell–surface interfaces. In this situation, the cells can be affected by electromagnetic forces or potentially extreme electrostatic interactions, which contrasts with the situations that prevail in vivo during neural development or nerve regeneration. The reason for this is that electrical currents in the body are mediated by the migration of ions and not electrons [93,94,95]. 

Although scaffold conductivity can play a significant role in improving the electrical induction process, recent studies have offered contradictory findings about the specific mechanisms of the impact of electrical stimulation on cultured cells. Grossemy et al. explained that the neuronal differentiation and neurite outgrowth of cultured cells on non-conductive scaffolds rely more on ion transport in the culture medium and the porosity of the scaffold fibers, and it can more closely imitate the natural conditions of nerve regeneration in vivo than using conductive surfaces, which is generally based on the electron current [96,97]. Multiple independent reactions can occur at the anode and cathode; when charged electrodes are placed in the liquid medium, electrochemical reactions at the electrodes generate ions. The ionic current intensity depends on different cases like the nature of the electrodes, the electrode potential, and the concentration and properties of the culture media contents. Therefore, this research emphasizes the importance of electrical signaling based on ion transfer through cell culture media and proved the importance of applying an external EF compared with the type of scaffold used. Their findings highlighted the significance of medium-based electrical phenomena in improving cell activity and pointed out that using electroactive platforms may not be critical for exploiting ES in cell culture [86]. 

It is worth mentioning that since we did not confirm the cell differentiation profile by immunocytochemistry or electrophysiological analysis, it is better to refer to the cells after ES treatment as neuron-like cells. During culture, the morphology and gene expression profile of the cells may be subject to substantial changes. While they may resemble neural cells, they may not display the functional features of mature neurons [98,99]. Furthermore, the mechanisms by which ES regulates neurite outgrowth and neural commitment are not fully understood, but several hypotheses have emerged to explain it. For instance, ES can cause the redistribution of ion channels and receptors of the plasma membrane and regulate intracellular signaling pathways, such as MAPK/ERKs, integrin, and PI3K/Akt, which trigger cytoskeletal reorganization and ultimately lead to cellular responses [34,100,101]. ES can be used synergistically along with other approaches, decreasing the cost and potentially alleviating some of the issues currently prevailing in NTE. The combination of specific biomaterial platforms and low-risk ES will offer numerous benefits over other inducers and allow for precise cellular regulation. 

## 4. Conclusions

We fabricated PCL/PLLA/SWNT nanofibrous composite scaffolds and investigated their physicochemical properties as potential candidates for NTE. The incorporation of SWNT effectively increased the conductivity of the scaffolds. The results revealed their cytocompatibility and nontoxic effects on PC12 cells and BMSCs. In addition, this study aimed to successfully culture cells on PCL/PLLA/SWNT scaffolds under ES without exogenous growth factors, while determining which of the two ES protocols (100 mV/cm for 10 min/day and 200 mV/cm for 20 min/day) was more efficient for neural differentiation in terms of upregulating the transcription of neural markers. The application of 200 mV/cm for 20 min/day to cells resulted in a significant increase in neural differentiation markers. Taken together, this study adds to the growing body of evidence supporting the use of ES to influence the neural commitment of progenitor cells. However, in order to validate these results, future research should focus on checking the expression of proteins and measuring the functional status of the cells using electrophysiological measurements.

## Figures and Tables

**Table 1:** List of genes, primer sequences, and melting temperatures (Tm) used in qPCR.

Gene Symbol	Gene Description	Primer Base Sequences (5′-3′)		Tm (°C)
		Forward	Reverse	
*NES*	Nestin	TGG AAC AGA GAT TGG AAG GC	CAG CAG AGT CCT GTA TGT AGC	58
*MAP-2*	Microtubule-associated protein 2	ACC AAC TCA TCT CTC CTG TG	GGT TAT TCC ATC AGT GAC TTT GT	57
*TUBB*	β Tubulin-3	TTT ATC TTC GGT CAG AGT GGT G	GGC AGT CAC AAT TCT CAC ATT C	58
*HPRT1*	Hypoxanthine phosphoribosyl transferase 1	CCA GCG TCG TGA TTA GTG	CGA GCA AGT CTT TCA GTC C	56

**Table 2:** Mechanical properties and electrical conductivity of electrospun PCL, PCL/PLLA, and PCL/PLLA/SWNT nanofibrous scaffolds.

Scaffold	Young’s Modulus (MPa)	Tensile Strength (MPa)	Volume Resistivity (μohm·cm)	Volume Conductivity (μS/cm)
PCL	1.510 ± 0.8	1.73 ± 0.3	29.6	0.0311
PCL/PLLA	73.50 ± 1.7	5.24 ± 0.4	30.1	0.0332
PCL/PLLA/SWNT	39.49 ± 2.3	4.53 ± 0.5	15.1	0.0663

## Data Availability

Raw data were generated at Department of Biotechnology, College of Science, University of Tehran. Derived data supporting the findings of this study are available from the corresponding authors.

## Supplementary Materials

The following supporting information can be downloaded at: https://www.mdpi.com/article/10.3390/bioengineering10121438/s1, Figure S1: Setup for electrical stimulation of cells. (**a**) Cells were seeded on PCL/PLLA/SWNT scaffolds attached to the bottom of a 12-well cell culture plate. Electrical stimulation was provided by custom-made stainless steel electrodes. (**b**,**c**) The multi-well plates were placed in an incubator and electrical stimulation was delivered via wires connected to a signal generator. (**d**) Example of the distribution of the experimental study groups on the multi-well plate and the electrical stimulation patterns (period, magnitude, and stimulation interval) for PC12 cells and BMSCs.
