# Supplementary material for: Electroconductive Nanofibrous Scaffolds Enable Neuronal Differentiation in Response to Electrical Stimulation without Exogenous Inducing Factors
Source: Bioengineering (Basel). 2023 Dec 18;10(12):1438. doi: 10.3390/bioengineering10121438 (PMC10740536; doi:10.3390/bioengineering10121438)
Supplement: Supplementary file 1 [file bioengineering-10-01438-s001.zip › bioengineering-2736202-supplementary.pdf]

Supplementary information

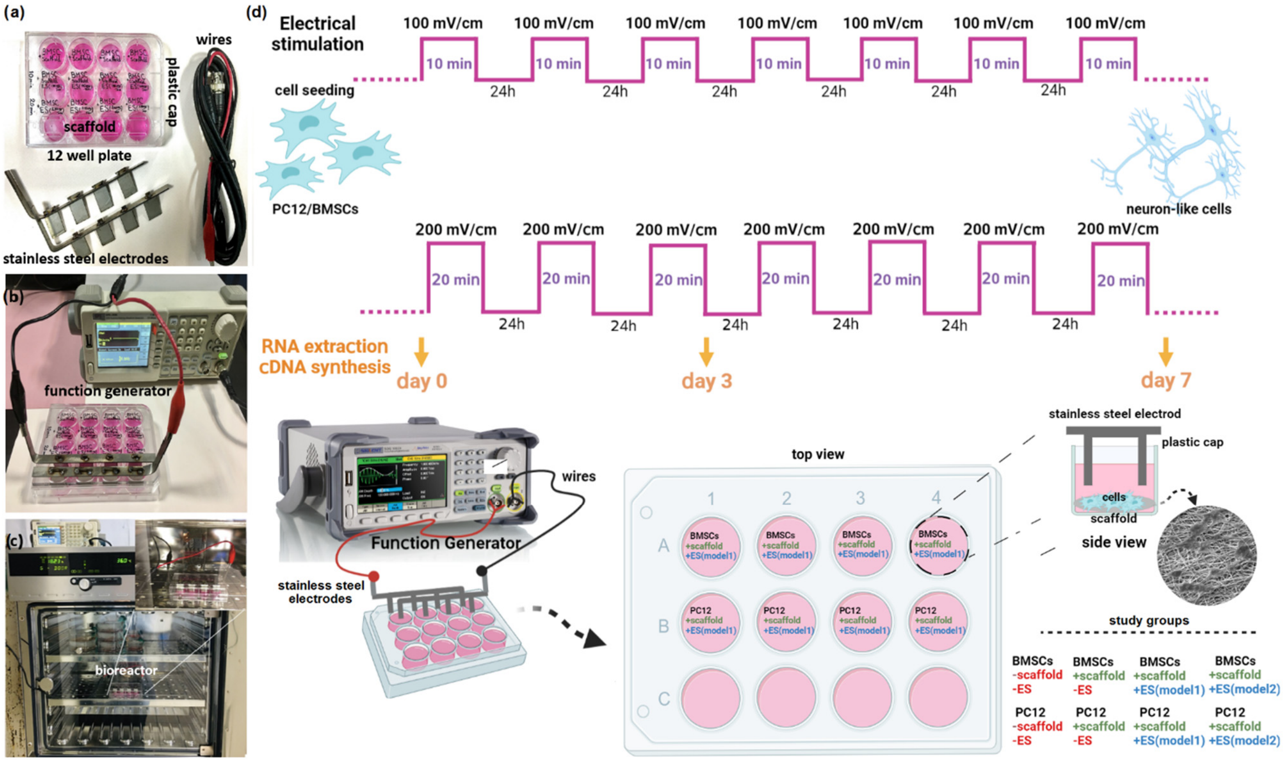

Figure S1. Setup for electrical stimulation of cells. (a) Cells were seeded on PCL/PLLA/SWNT scaffolds attached to the bottom of a 12-well cell culture plate. Electrical stimulation was provided by custom-made stainless steel electrodes. (b,c) The multi-well plates were placed in an incubator and electrical stimulation was delivered via wires connected to a signal generator. (d) Example of the distribution of the experimental study groups on the multi-well plate and the electrical stimulation patterns (period, magnitude, and stimulation interval) for PC12 cells and BMSCs.
